# Burden of Medically Attended Diarrhea and Outpatient *Clostridioides difficile* Infection Among Persons in 2 Large Integrated Healthcare Settings, 2016–2021

**DOI:** 10.1093/ofid/ofad680

**Published:** 2024-01-11

**Authors:** Sara Y Tartof, Mark A Schmidt, Richard Contreras, Frederick J Angulo, Ana Florea, Joanna L Barreras, Judy Donald, Joann Zamparo, Deborah Ling Grant, Elizabeth Shuster, Elisa Gonzalez, Jennifer L Kuntz

**Affiliations:** Department of Research and Evaluation, Kaiser Permanente Southern California, Pasadena, California, USA; Department of Health Systems Science, Kaiser Permanente Bernard J. Tyson School of Medicine, Pasadena, California, USA; Department of Health Systems Science, Kaiser Permanente Bernard J. Tyson School of Medicine, Pasadena, California, USA; Science Programs Department, Kaiser Permanente Northwest Center for Health Research, Portland, Oregon, USA; Department of Research and Evaluation, Kaiser Permanente Southern California, Pasadena, California, USA; Vaccines, Antivirals, and Evidence Generation, Pfizer Inc, NewYork, New York, USA; Department of Research and Evaluation, Kaiser Permanente Southern California, Pasadena, California, USA; Department of Research and Evaluation, Kaiser Permanente Southern California, Pasadena, California, USA; Science Programs Department, Kaiser Permanente Northwest Center for Health Research, Portland, Oregon, USA; Vaccines, Antivirals, and Evidence Generation, Pfizer Inc, NewYork, New York, USA; Department of Research and Evaluation, Kaiser Permanente Southern California, Pasadena, California, USA; Science Programs Department, Kaiser Permanente Northwest Center for Health Research, Portland, Oregon, USA; Vaccines, Antivirals, and Evidence Generation, Pfizer Inc, NewYork, New York, USA; Science Programs Department, Kaiser Permanente Northwest Center for Health Research, Portland, Oregon, USA

**Keywords:** *Clostridioides difficile*, diarrhea, incidence, Kaiser Permanente, real-world

## Abstract

**Background:**

Identification of *Clostridioides difficile* infection (CDI) in the community setting is increasing. We describe testing for CDI among patients with medically attended diarrhea (MAD) in the outpatient setting, and the incidence of outpatient CDI.

**Methods:**

This was a retrospective cohort study among members ≥18 years of age from Kaiser Permanente Southern California and Kaiser Permanente Northwest from 1 January 2016 through 31 December 2021. MAD was identified by outpatient diarrheal *International Classification of Diseases, Tenth Revision* diagnosis codes, and CDI through positive laboratory results. Outpatient CDI was defined by no hospitalization ≤7 days after specimen collection. Incidence rates (IRs) of outpatient CDI were stratified by select demographic and clinical variables. Outpatient CDI burden 12 months following index date was measured by CDI-associated healthcare visits, and CDI testing and treatment.

**Results:**

We identified 777 533 MAD episodes; 12.1% (93 964/777 533) were tested for CDI. Of those tested, 10.8% (10 110/93 964) were positive. Outpatient CDI IR was 51.0 (95% confidence interval [CI], 49.8–52.2) per 100 000 person-years, decreasing from 58.2 (95% CI, 55.7–60.7) in 2016 to 45.7 (95% CI, 43.7–47.8) in 2021. Approximately 44% (n = 4200) received an antibiotic 30 days prior to index date and 84.1% (n = 8006) CDIs were “community-associated” (no hospitalizations 12 weeks prior to index date). Of outpatient CDIs, 6.7% (n = 526) had a CDI-associated hospitalization ≤12 months.

**Conclusions:**

There was a high incidence of outpatient CDI despite infrequent CDI testing among patients with MAD. The majority of those with outpatient CDI had no recent antibiotic use and no recent hospitalization. Further studies are needed to understand the source and management of medically attended outpatient CDI.

## Teaser

A high incidence of outpatient *Clostridioides difficile* infection (CDI) was detected despite infrequent CDI testing among patients with medically attended diarrhea. The majority of those with outpatient CDI had no recent antibiotic use and no recent hospitalization.

*Clostridioides difficile* infection (CDI) can result in a mild to severe diarrheal illness and is a leading cause of infectious diarrhea. CDI causes substantial morbidity and mortality in the United States, where it is one of the most common healthcare-associated infections [1]. In 2017, the Centers for Disease Control and Prevention (CDC) estimated that there were 462 100 laboratory-confirmed cases of CDI occurring in the United States, resulting in 223 900 hospitalizations and 12 800 deaths per year [2, 3].

According to CDC population-based surveillance of laboratory-confirmed CDI, the incidence of community-associated CDI cases increased from 57.8 per 100 000 population in 2014 to 63.3 per 100 000 population in 2019, while the proportion of laboratory-confirmed CDI cases that were community-associated increased from 40.8% in 2014 to 52.2% in 2019 [4, 5].

Despite the high disease burden of CDI, it is likely that many other CDI cases occur among persons in the community but are not laboratory confirmed. Undiagnosed CDI cases in the community, both healthcare-associated and community-associated, can be the result of a person with diarrhea not seeking medical care, or missed opportunities for stool specimen collection and CDI testing among persons who seek medical care for diarrhea. The incidence of medically attended diarrhea (MAD) among persons in the community and the frequency at which such persons with MAD are tested for CDI has not been well described. Therefore, little is known about the extent of undiagnosed medically attended CDI among persons in the community. The CDC Emerging Infections Program population-based surveillance reported that among those 55 years and older, 38%–48% of community-associated laboratory-confirmed CDI cases were hospitalized within 6 days of specimen collection in 2017, suggesting that the majority of laboratory-confirmed CDI cases in older adults occur among outpatients [6]. However, the healthcare utilization and health outcomes of the patients with medically attended CDI who are not subsequently hospitalized (ie, outpatient CDI cases) have not been well described.

Our objectives were to describe the population-based incidence of MAD and testing for *C difficile* in the outpatient setting, the population-based incidence of outpatient CDI, and healthcare utilization outcomes among patients with outpatient CDI.

## METHODS

### Study Design

We conducted our study among adult (≥18 years of age) members of Kaiser Permanente Southern California (KPSC) and Kaiser Permanente Northwest (KPNW) between 1 January 2016 and 31 December 2021. KPSC and KPNW are integrated healthcare systems serving diverse populations of >4.7 million and >600 000 members, respectively. Both systems employ electronic health records that integrate clinical data including diagnostic, pharmacy, laboratory, and vaccination history information across all settings of care. Care delivered to members outside of the Kaiser Permanente (KP) system is also captured through claims submitted to KP for reimbursement.

### Identification of Medically Attended Diarrhea and *C difficile* Laboratory Testing in the Outpatient Setting

We identified MAD through *International Classification of Diseases, Tenth Revision* (*ICD-10*) diagnosis codes related to diarrheal illness recorded on outpatient healthcare encounters, including ambulatory, urgent care, emergency department, telephone, and video visits. (Supplementary Table 1). To be included in these analyses, we required that members have at least 1 day of health plan membership during the study period. We defined the index date of a MAD episode as the date of an initial MAD diagnosis and the end date as the date on which a patient had no further MAD diagnosis codes for 55 days. We allowed patients to have >1 MAD episode, if the episodes were separated by at least 56 days. For these MAD episodes, we assessed the occurrence of laboratory orders and testing, including for *C difficile* (Supplementary Table 2), during the time between the start and end dates of a MAD episode.

### Identification of Outpatient *C difficile* Infection

For analyses focused on incidence and burden of outpatient CDI, we required members to have ≥12 months of continuous enrollment (allowing for 45-day administrative gaps) prior to CDI, to allow for capture of comorbidities and other variables evaluated in the baseline period. We identified CDI episodes through positive *C difficile* laboratory test results. At KPSC and KPNW, laboratory testing for CDI includes enzyme-linked immunoassay assays for glutamate dehydrogenase antigen and cytotoxins (toxin A or toxin B), with polymerase chain reaction testing for the toxin gene reflexively done if necessary. We defined the index date for an outpatient CDI as the date of specimen collection related to a positive laboratory test. We defined the end date of an outpatient CDI episode as the date occurring 55 days after the last evidence of CDI (eg, positive laboratory test or CDI-coded encounters).

We defined an incident outpatient CDI case as a positive CDI laboratory test without hospitalization on the date of stool specimen collection or within the subsequent 7 days following index date and without evidence (laboratory or diagnostic) of an ongoing *C difficile* episode within the previous 56 days. For calculation of incidence rates (IRs), we allowed individuals to have multiple, incident outpatient CDI episodes provided there was a gap of at least 56 days between evidence of CDI and a subsequent positive *C difficile* test.

### Healthcare Utilization Following Outpatient *C difficile* Infection

We measured burden of healthcare utilization among patients with outpatient CDI in the 12 months following index date. We measured healthcare utilization through the number of outpatient visits, number of virtual visits, number of emergency department visits, hospitalization (yes/no), and number of hospitalizations with a CDI diagnosis code following the index date. We measured non-CDI and CDI-related healthcare utilization and defined “CDI-related” healthcare visits by the presence of *ICD-10* code A04.7. We identified additional *C difficile* laboratory testing in the 1–14 days, 15–56 days, 57–180 days, and 181–365 days after index date. We identified treatment for CDI through metronidazole, oral vancomycin, fidaxomicin, or rifaximin dispensings at KP outpatient pharmacies during the initial CDI episode (days 1–56 post–index date) and in the 57–180 days and 181–365 days after index date. We also collected the number of CDI treatment dispensings in the 365 days after index date.

### Characteristics of Patients With Outpatient *C difficile* Infection

We collected clinical and demographic variables for patients with MAD and CDI episodes, including age at index date, sex (male, female), race/ethnicity (non-Hispanic White, Hispanic, non-Hispanic Black, non-Hispanic Asian, other or Pacific Islander, unknown), number of Charlson Comorbidity Index (CCI) conditions (Supplementary Table 3) [7, 8], presence of individual CCI conditions (myocardial infarction, congestive heart failure, peripheral vascular disease, cerebrovascular disease, dementia, chronic pulmonary disease, rheumatologic disease, peptic ulcer disease, liver disease [mild, moderate, severe], diabetes [with or without complications], hemiplegia or paraplegia, renal disease, malignancy, including leukemia or lymphoma, metastatic solid tumor, human immunodeficiency virus/AIDS), inpatient and outpatient antibiotic use in the 30 days prior to index date (any antibiotic, aminoglycosides, carbapenems, cephalosporins, clindamycin, fluoroquinolones, glycylcyclines, macrolides, monobactam, penicillins, polymyxin, sulfonamides, tetracyclines), and inpatient and outpatient healthcare utilization 1 year prior to index date. We further classified outpatient CDI cases as being healthcare-associated or community-associated. We defined community-associated CDI as an outpatient CDI without documentation of a hospital admission in the 12 weeks (84 days) prior to index date, and healthcare-associated CDI as outpatient CDI with documentation of a hospital admission in the 12 weeks (84 days) prior to index date.

### Statistical Analysis

We describe frequency of MAD episodes and proportion of episodes with laboratory testing. Of MAD episodes positive for *C difficile*, we describe the number of encounters and duration of CDI episodes. We calculated the population-based incidence of outpatient CDI among KPSC and KPNW members as the number of outpatient CDI episodes per 100 000 person-years (PY). We calculated IRs and exact Poisson 95% confidence intervals (CIs) for the overall population and by age group, calendar year, sex, race/ethnicity, Charlson comorbidity, and site (KPSC, KPNW). Descriptive analyses, including mean and range, median, and summary counts and percentages are presented for counts of CDI-related healthcare visits, CDI testing, and CDI treatment in the 12 months following first outpatient CDI among participants with 1 year of membership following index date. All analyses were conducted using SAS version 9.4 software.

### Patient Consent Statement

The study protocol was reviewed and approved by the KPNW Institutional Review Board, which waived requirement of informed consent (FWA000002344; study number 12882; approval date: 24 August 2021). The KPSC Institutional Review Board ceded to KPNW.

## RESULTS

From 2016 to 2021, we identified 777 533 MAD episodes among 592 877 individuals (Table 1). During the 6-year study, we estimate that 8.5% of the total KPSC and KPNW membership (n = 6 941 823) experienced at least 1 MAD episode. Persons with at least 1 MAD episode had a mean age of 48.1 years, were majority female (61.4%), and were most commonly Hispanic (40.6%) or non-Hispanic White (39.5%) (Table 2). Among persons with a MAD episode, 11.9% (n = 70 580) received antibiotics in the previous 30 days and 9.8% (n = 58 344) had been hospitalized in the prior year.

**Table 1.** Occurrence and Testing of Medically Attended Diarrhea and Medically Attended *Clostridioides difficile* Infection Episodes, 2016–2021

Episodes	No. (%)
Total MAD episodes^a^	n = 777 533
Laboratory testing related to MAD	
Laboratory testing ordered by provider (yes)	287 906 (37.0)
Laboratory test completed (yes)	180 302 (23.2)
*C difficile testing* and laboratory confirmation	
*C difficile* laboratory test ordered by provider (yes)	93 964 (12.1)
Positive *C difficile* laboratory test (% of MAD episodes positive for *C difficile*)	10 110 (1.3)
Positive laboratory test for *C difficile* and another pathogen (ie, coinfection)	7369 (0.95)
Positive laboratory test for a non–*C difficile* pathogen	163 823 (21.1)
Not laboratory confirmed	610 969 (78.6)
Total CDI episodes	n = 10 110
No. of CDI encounters per CDI episode	
1	4043 (40.0)
2–5	5231 (51.7)
≥6	836 (8.3)
Duration of CDI episode	
<56 d	4043 (40.0)
56–180 d	5798 (57.4)
181–365 d	254 (2.5)
>365	15 (0.1)
Hospitalization within 7 d following index date for CDI episode (yes)	477 (4.7)

Abbreviations: CDI, *Clostridioides difficile* infection; MAD, medically attended diarrhea.

^a^Among patients with Kaiser Permanente membership for ≥1 day at some point during study period.

**Table 2.** Demographic and Clinical Characteristics of Individuals With at Least 1 Outpatient *Clostridioides difficile* Infection Episode, 2016–2021

Characteristic	CDI (n = 9517)^a^
Age, y, mean (SD)	60.9 (18.6)
Age, y, median (IQR)	64 (27)
Age, y	
18–49	2523 (26.5)
50–54	673 (7.1)
55–59	832 (8.7)
60–64	937 (9.9)
65–69	1003 (10.5)
70–74	1115 (11.7)
75–79	861 (9.1)
80–84	725 (7.6)
≥85	848 (8.9)
Sex, female	6030 (63.4)
Race/ethnicity	
Non-Hispanic Asian	483 (5.1)
Non-Hispanic Black	691 (7.2)
Hispanic	2648 (27.8)
Pacific Islander/Other	173 (1.8)
Unknown	63 (0.7)
Non-Hispanic White	5459 (57.4)
Count of CCI conditions in past year	
Overall, mean (SD)	2.4 (2.9)
Preexisting medical conditions (year prior)	
Myocardial infarction	739 (7.8)
Congestive heart failure	1093 (11.5)
Peripheral vascular disease	3293 (34.6)
Cerebrovascular disease	819 (8.6)
Dementia	566 (6.0)
Chronic pulmonary disease	2302 (24.2)
Rheumatologic disease	415 (4.4)
Peptic ulcer disease	274 (2.9)
Any liver disease	316 (3.3)
Diabetes mellitus	2292 (24.1)
Hemiplegia or paraplegia	192 (2.0)
Renal disease	2245 (23.6)
Malignancy, including leukemia or lymphoma	1104 (11.6)
Metastatic solid tumor	372 (3.9)
HIV/AIDS	28 (0.3)
Antibiotic use in 30 d prior	
Any antibiotic use	4200 (44.1)
Aminoglycosides	65 (0.7)
Carbapenems	36 (0.4)
Cephalosporins	1801 (18.9)
Clindamycin	718 (7.5)
Fluoroquinolones	1341 (14.1)
Glycylcyclines	0 (0)
Macrolides	233 (2.5)
Monobactam	9 (0.1)
Penicillins	1260 (13.2)
Polymyxin	0 (0)
Sulfonamides	249 (2.6)
Tetracyclines	116 (1.2)
No. of diarrhea/CDI episodes	
1	8772 (92.2)
2	611 (6.4)
≥3	134 (1.4)
Hospitalization in past year	3337 (35.1)
Hospitalization in prior 30 d	662 (7.0)
Hospitalization in prior 31–84 d	849 (8.9)
Hospitalization in prior 85–180 d	681 (7.2)
Hospitalization in prior 181–365 d	1145 (12.0)
Total days of hospitalization in past year (among those hospitalized), mean (SD)	9.9 (15.2)
No. of outpatient visits in past year, median (IQR)	15 (19)
Classification of CDI	
Healthcare associated	1511 (15.9)
Community associated	8006 (84.1)

Abbreviations: CCI, Charlson Comorbidity Index; CDI, *Clostridioides difficile* infection; HIV, human immunodeficiency virus; IQR, interquartile range; SD, standard deviation.

^a^Required at least 1 year of Kaiser Permanente membership before CDI; not hospitalized within 7 days of index date.

Stool specimen testing was ordered for 37% (287 906/777 533) of all MAD episodes (Table 1) and 93 964 (12.1%) of all MAD episodes were tested for *C difficile*; among those, 11% (n = 10 110) were positive for *C difficile*. Testing for *C difficile* was most frequent among patients aged ≥70 years (20.7% of MAD episodes in this group; n = 28 480) and was least common for younger patients aged 18–49 years (8.3% [n = 34 034]) (Supplementary Table 4). Except for those with unknown race/ethnicity, disparities by race/ethnicity in testing for CDI were generally not observed in older ages but were observed more among younger age groups (Supplementary Table 4). The majority of these medically attended CDI episodes had a duration of 56–180 days (57.4% [n = 5798]) and included 2 to 5 healthcare visits (51.7% [n = 5231]). A small percentage of medically attended CDI episodes (4.7% [n = 477]) resulted in hospitalization within 7 days of index date.

We identified 9517 individuals who met membership criteria and had at least 1 medically attended outpatient CDI episode, defined as no hospitalization within 7 days following the index date. The mean age of patients with outpatient CDI was 60.9 years and the majority were female (63.4%) (Table 2). The majority of patients were non-Hispanic White (57.4% [n = 5459]), followed by Hispanic (27.8% [n = 2648]) and Non-Hispanic Black (7.2% [n = 691]). Less than one-half of patients with outpatient CDI (44.1%) received an antibiotic in the 30 days before the index date of their first CDI episode. The most commonly used antibiotic classes among outpatient CDI patients were cephalosporins (18.9% [n = 1801]), fluoroquinolones (14.1% [n = 1341]), and penicillins (13.2% [n = 1260]). Approximately one-third of patients with outpatient CDI had been hospitalized in the previous year (35.1% [n = 3337]). The majority (84.1% [n = 8006]) of first episodes of outpatient CDI were “community associated.” While the majority (92.2% [n = 8772]) of persons with outpatient CDI had a single CDI episode during the study time period, roughly 6.4% of patients (n = 611) had 2 episodes and 1.4% (n = 134) had 3 or more episodes.

The incidence of outpatient CDI from 2016 to 2021 was 51.0 (95% CI, 49.8–52.2) cases per 100 000 PY. The incidence of outpatient CDI decreased from 58.2 (95% CI, 55.7–60.7) cases per 100 000 PY in 2016 to 45.7 (95% CI, 43.7–47.8) cases per 100 000 PY in 2021 (Table 3). The rate of CDI testing among MAD episodes did not similarly decrease over the study period, with the exception of a drop in 2020, and lower rates in 2021 compared to rates in 2016–2019 (Supplementary Table 5). Incidence of medically attended outpatient CDI was higher among females than males (58.1 [95% CI, 56.7–59.5] vs 37.3 [95% CI, 36.2–38.5] cases per 100 000 PY), and increased with age, from 25.7 (95% CI, 24.9–26.6) per 100 000 PY among those 18–49 years old to 228.1 (95% CI, 209.8–247.6) per 100 000 PY among those 85 years and older. The medically attended outpatient CDI IR per 100 000 PY was highest for White patients (70.8 [95% CI, 69.0–72.6]), followed by Black patients (46.0 [95% CI, 42.8–49.5]) and Pacific Islander/other patients (44.4 [95% CI, 38.6–50.9]). IRs were high among patients with comorbidities, such as those with congestive heart failure (299.5 [95% CI, 273.7–327.0]), hemiplegia or paraplegia (285.4 [95% CI, 226.6–354.7]), dementia (260.7 [95% CI, 217.7–309.8]), and peptic ulcer disease (255.2 [95% CI, 207.7–310.4]).

**Table 3.** Incidence of Outpatient *Clostridioides difficile* Infection Episodes^a^, 2016–2021

Characteristic	Overall		
	No.	Person-years	Incidence (95% CI) per 100 000 PY
Overall	10 545	21 890 314	48.2 (47.3–49.1)
Year^b^			
2016	2104	3 616 550	58.2 (55.7–60.7)
2017	1929	3 780 198	51 (48.8–53.4)
2018	2084	3 927 373	53.1 (50.8–55.4)
2019	1885	4 005 216	47.1 (45.0–49.2)
2020	1673	4 095 522	40.8 (38.9–42.9)
2021	1888	4 132 314	45.7 (43.7–47.8)
Sex			
Male	3910	10 469 081	37.3 (36.2–38.5)
Female	6635	11 421 233	58.1 (56.7–59.5)
Age, y			
18–49	3323	12 919 050	25.7 (24.9–26.6)
50–54	853	1 909 769	44.7 (41.7–47.8)
55–59	1005	1 864 911	53.9 (50.6–57.3)
60–64	1020	1 613 981	63.2 (59.4–67.2)
65–69	1256	1 466 716	85.6 (81.0–90.5)
70–74	1027	919 582	111.7 (105.0–118.7)
75–79	813	590 652	137.6 (128.3–147.4)
80–84	674	355 693	189.5 (175.5–204.4)
≥85	574	251 635	228.1 (209.8–247.6)
Race/ethnicity			
White	5993	8 464 647	70.8 (69.0–72.6)
Black	725	1 574 447	46 (42.8–49.5)
Asian	512	2 195 909	23.3 (21.3–25.4)
Hispanic	2942	7 584 478	38.8 (37.4–40.2)
Pacific Islander/Other	207	465 827	44.4 (38.6–50.9)
Unknown	166	1 606 827	10.3 (8.8–12.0)

Abbreviations: CI, confidence interval.

^a^Outpatient *Clostridioides difficile* episode defined patient seeking medical care with diarrhea with a positive *C difficile* laboratory test (1) without hospitalization on the date of stool specimen or within the subsequent 7 days; and (2) without evidence (laboratory or diagnostic) of an ongoing *C difficile* episode within the previous 56 days.

^b^Counts from individual years sum to greater than column total as participants can have CDI episodes in >1 year.

CDI-associated healthcare utilization in the 12 months following first outpatient CDI episode was low to moderate for ambulatory care (virtual, outpatient, and emergency department visits), and 6.7% (n = 526) of outpatient CDI patients had a CDI-associated hospitalization in the 8 days to 1 year following the index date (Table 4). Roughly one-third of outpatient CDI patients underwent repeat CDI testing in the year following the index date (32.9% [n = 2566]), with the majority of subsequent testing occurring >2 weeks after their initial *C difficile* test. The majority of outpatient CDI cases (72.4% [n = 5653]) received CDI treatment, the majority of which (97.2%) was dispensed within 56 days of the index date.

**Table 4.** Outcomes in the 12 Months Following First Diagnosis of *Clostridium difficile* Infection Episode, 2016–2021

Outcome	CDI (n = 7807^a^)
Healthcare utilization	
Virtual CDI-associated visits, mean (range), median	0.3 (0–13), 0
Outpatient CDI-associated visits, mean (range), median	0.6 (0–134), 0
Emergency CDI-associated department visits, mean (range), median	0.2 (0–13), 0
Any CDI-associated hospitalization, No. (%)	526 (6.7)
*C difficile* laboratory testing	
Any *C difficile* testing in 365 d after index date, No. (%)	2566 (32.9)
Timing of *C difficile* testing among those tested, No. (%)	
1–14 d after index date	151 (5.9)
15–56 d after index date	1376 (53.6)
57–180 d after index date	709 (27.6)
181–365 d after index date	330 (12.9)
Treatment for CDI	
Outpatient treatment dispensing, No. (%)	
Any treatment^b^	5653 (72.4)
Vancomycin	4820 (61.7)
Metronidazole	1107 (14.8)
Fidaxomicin	382 (4.9)
Timing of first treatment, among those treated, No. (%)	
Same day of index date	713 (12.6)
1–56 d after index date	4784 (84.6)
57–180 d after index date	111 (2.0)
181–365 d after index date	45 (0.8)
No. of treatment dispensings, mean (SD)	2.0 (1.8)

Abbreviations: CDI, *Clostridioides difficile* infection; SD, standard deviation.

^a^Individuals required at least 1 year of membership after *C difficile* index date.

^b^Includes metronidazole, oral vancomycin, fidaxomicin.

## DISCUSSION

Our study found a high incidence of outpatient CDI (51.0/100 000 PY) from 2016 to 2021. The high outpatient CDI incidence is noteworthy and likely an underestimate of the true burden of outpatient CDI, given the low frequency (12%) of CDI testing among patients with MAD. Consistent with population-based studies of CDI incidence, the incidence of outpatient CDI was higher among females, non-Hispanic White members, and those with underlying comorbid conditions, and incidence increased with age. We also found that the majority of outpatient CDI cases occurred among people without a recent history of antibiotic use or hospitalization. Finally, the majority of patients did not utilize high levels of ambulatory healthcare related to CDI following their diagnosis. Less than 10% experienced CDI-associated hospitalization following an episode, and 30% of patients did not receive treatment for their CDI at the time of their initial diagnosis, suggesting lower severity of CDI diagnosed and managed in the outpatient setting.

Prior work has demonstrated that females (vs males) and individuals of a White racial/ethnic background (vs other race/ethnicities) are more likely to seek care for a health problem, which may contribute to our findings of high incidence of CDI among non-Hispanic White females [9, 10]. Our study also found that 84.1% of first episodes of outpatient CDI were community-associated CDI, having no hospitalization in the previous 12 weeks. Last, in individuals with at least 1 CDI episode, our study found peripheral vascular disease, chronic pulmonary disease, diabetes, and renal diseases to be the most prevalent conditions. Previous studies have found an association between cardiac disease, chronic kidney disease, diabetes, and inflammatory bowel disease and community-associated CDI [11, 12]. Thus, given the higher prevalence of these comorbidities in our study population, KP members who present with diarrhea and also have 1 of these comorbidities may benefit from CDI testing.

Furthermore, 56% of patients with outpatient CDI in our study did not have a history of antibiotic use in the preceding month. Despite prior use of clindamycin, fluoroquinolones, and carbapenems being the most likely to be associated with CDI [13, 14], our study found a high rate of prior use of cephalosporins (18.9%), fluoroquinolones (14.1%), and penicillins (13.2%) in patients with outpatient CDI, whereas the rates of prior use of clindamycin and carbapenems were much lower (7.5% and 0.4%, respectively), which reflect KP prescribing practices. Furthermore, only 35% of patients with outpatient CDI had a history of hospitalization in the 1 year prior to their diagnosis. Our findings are consistent with other studies reporting a lack of a history of healthcare facility or antibiotic use exposures among patients presenting with CDI in the community [13, 15, 16]. However, due to our shorter lookback period for antibiotic use (30 days) compared to other studies, and a low but existing possibility that patients may have received antibiotics in non-KP settings, it may be possible some previous antibiotic use was missed. Nonetheless, given these similar observations across studies, it is reasonable to assume that there are reservoirs of *C difficile* beyond healthcare facilities and risk factors beyond antibiotic use contributing to the occurrence of CDI in the general community. Some studies have reported *C difficile* transmission within households [17, 18] and between humans and domestic or farm animals [19–21], and the possibility of foodborne transmission has also been suggested [22]; however, further research is needed to determine whether or to what extent these sources are contributing to the current burden of CDI in the community.

We found that approximately one-third of patients with outpatient CDI did not receive treatment in the year following their infection and, in the 1 year following their diagnosis, did not have frequent contact with the healthcare system. The reasons for these observations cannot be fully explained by this study; however, it could be hypothesized that outpatient CDI cases may be less severe and more likely to resolve without antibiotic treatment. Additional research is needed to evaluate the severity of illness, particularly the impact on quality of life, in patients with outpatient CDI. There is also the potential for undertreatment or, in cases where CDI was detected through a laboratory test that looks for multiple gastrointestinal pathogens, there is the possibility that positive *C difficile* test results did not receive adequate follow-up, leading to no treatment initiation by providers or a lack of understanding of the need to adhere to prescribed treatment by patients.

Our study has a number of limitations. First, we were unable to completely capture a history of long-term care facility or nursing home stays, so it is possible that some outpatient CDI cases occurred among patients within those settings and that some cases classified as “community associated” actually had a history of a stay in a healthcare facility in the weeks and months preceding their CDI diagnosis. Next, we utilized positive *C difficile* laboratory testing for case identification. Patients may experience CDI symptoms and may have CDI without seeking healthcare or obtaining testing, leading to underestimation of the true burden of outpatient CDI. Lack of healthcare seeking for outpatient CDI would have been the most pronounced during the period from March 2020 through December 2021, when the coronavirus disease 2019 pandemic dramatically altered clinical services, particularly ambulatory care. This was reflected in lower rates of CDI testing among MAD episodes in 2020 and 2021, and it is likely that our IR estimates during these years are particularly underestimated. Finally, although our study shows that patients with outpatient CDI do not utilize high levels of healthcare, our study could not directly measure illness severity or the impact of CDI on their quality of life. Despite these limitations, our study has a number of strengths. Unlike prior studies of CDI that are primarily focused on case identification in the outpatient setting [23, 24], our study was conducted in 2 integrated healthcare systems, which allowed for identification of CDI cases in the outpatient setting and the ability to track patients’ healthcare utilization and risk factor history as well as the clinical course of their infection.

In summary, we detected a high incidence of outpatient CDI in 2 KP integrated healthcare systems. Notably, the majority of cases were community associated and occurred among people without a recent history of hospitalization or antibiotic use. While lower than expected rates of CDI treatment and relatively low levels of healthcare utilization in the 1 year following outpatient CDI diagnosis indicate short illness duration and limited impact on the healthcare system, the low frequency of *C difficile* testing among patients presenting with diarrhea indicates that outpatient CDI may be underdiagnosed and that the incidence of outpatient CDI may be higher than appreciated. Further studies on the quality-of-life impact of outpatient CDI on patients would be helpful to judge the disease burden of outpatient CDI. Furthermore, the high incidence of outpatient CDI may contribute to transmission within households or other environments or may predispose high-risk patients with comorbidities to subsequent CDI infections in the event of an immunocompromising event or medication.

## Supplementary Material

ofad680_Supplementary_Data

## Supplementary Data

Supplementary materials are available at *Open Forum Infectious Diseases* online. Consisting of data provided by the authors to benefit the reader, the posted materials are not copyedited and are the sole responsibility of the authors, so questions or comments should be addressed to the corresponding author.

## Notes

***Acknowledgments.*** We thank Lindsay Jenkins and Suzanne B. Salas for programming efforts in support of this study.

***Data availability.*** Anonymized data that support the findings of this study may be made available from the investigative team in the following conditions: (1) agreement to collaborate with the study team on all publications; (2) provision of external funding for administrative and investigator time necessary for this collaboration; (3) demonstration that the external investigative team is qualified and has documented evidence of training for human subjects protections; and (4) agreement to abide by the terms outlined in data use agreements between institutions.

***Financial support*.** This study was sponsored by Pfizer Inc. The study design was developed by KPSC and KPNW but approved by Pfizer. KPSC and KPNW collected and analyzed the data. Pfizer did not participate in the collection or analysis of data. KPSC, KPNW, and Pfizer participated in the interpretation of data, in the writing of the report, and in the decision to submit the paper for publication.
