# Supplementary material for: Burden of Medically Attended Diarrhea and Outpatient Clostridioides difficile Infection Among Persons in 2 Large Integrated Healthcare Settings, 2016–2021
Source: Open Forum Infect Dis. 2024 Jan 11;11(1):ofad680. doi: 10.1093/ofid/ofad680 (PMC10798856; doi:10.1093/ofid/ofad680)
Supplement: ofad680_Supplementary_Data [file ofad680_supplementary_data.docx]

**Supplement**

## Table S1. Medically-Attended Diarrhea Diagnostic Codes

| **Diagnosis Code** | **Diagnosis Description** |
| --- | --- |
| A00.0 | A00.0 (CHOLERA DUE TO VIBRIO CHOLERAE 01, BIOVAR CHOLERAE) |
| A00.1 | A00.1 (CHOLERA DUE TO VIBRIO CHOLERAE 01, BIOVAR ELTOR) |
| A00.9 | A00.9 (CHOLERA, UNSPECIFIED) |
| A01.00 | A01.00 (TYPHOID FEVER, UNSPECIFIED) |
| A02.0 | A02.0 (SALMONELLA ENTERITIS) |
| A02.1 | A02.1 (SALMONELLA SEPSIS) |
| A02.8 | A02.8 (OTHER SPECIFIED SALMONELLA INFECTIONS) |
| A02.9 | A02.9 (SALMONELLA INFECTION, UNSPECIFIED) |
| A03.0 | A03.0 (SHIGELLOSIS DUE TO SHIGELLA DYSENTERIAE) |
| A03.3 | A03.3 (SHIGELLOSIS DUE TO SHIGELLA SONNEI) |
| A03.8 | A03.8 (OTHER SHIGELLOSIS) |
| A03.9 | A03.9 (SHIGELLOSIS, UNSPECIFIED) |
| A04.0 | A04.0 (ENTEROPATHOGENIC ESCHERICHIA COLI INFECTION) |
| A04.1 | A04.1 (ENTEROTOXIGENIC ESCHERICHIA COLI INFECTION) |
| A04.2 | A04.2 (ENTEROINVASIVE ESCHERICHIA COLI INFECTION) |
| A04.3 | A04.3 (ENTEROHEMORRHAGIC ESCHERICHIA COLI INFECTION) |
| A04.4 | A04.4 (OTHER INTESTINAL ESCHERICHIA COLI INFECTIONS) |
| A04.5 | A04.5 (CAMPYLOBACTER ENTERITIS) |
| A04.6 | A04.6 (ENTERITIS DUE TO YERSINIA ENTEROCOLITICA) |
| A04.7 | A04.7 (ENTEROCOLITIS DUE TO CLOSTRIDIUM DIFFICILE) |
| A04.71 | A04.71 (ENTEROCOLITIS DUE TO CLOSTRIDIUM DIFFICILE, RECURRENT) |
| A04.72 | A04.72 (ENTEROCOLITIS DUE TO CLOSTRIDIUM DIFFICILE, NOT SPECIFIED AS RECURRENT) |
| A04.8 | A04.8 (OTHER SPECIFIED BACTERIAL INTESTINAL INFECTIONS) |
| A04.9 | A04.9 (BACTERIAL INTESTINAL INFECTION, UNSPECIFIED) |
| A05.0 | A05.0 (FOODBORNE STAPHYLOCOCCAL INTOXICATION) |
| A05.1 | A05.1 (BOTULISM FOOD POISONING) |
| A05.3 | A05.3 (FOODBORNE VIBRIO PARAHAEMOLYTICUS INTOXICATION) |
| A05.4 | A05.4 (FOODBORNE BACILLUS CEREUS INTOXICATION) |
| A05.9 | A05.9 (BACTERIAL FOODBORNE INTOXICATION, UNSPECIFIED) |
| A06.0 | A06.0 (ACUTE AMEBIC DYSENTERY) |
| A06.1 | A06.1 (CHRONIC INTESTINAL AMEBIASIS) |
| A06.2 | A06.2 (AMEBIC NONDYSENTERIC COLITIS) |
| A06.9 | A06.9 (AMEBIASIS, UNSPECIFIED) |
| A07.0 | A07.0 (BALANTIDIASIS) |
| A07.1 | A07.1 (GIARDIASIS [LAMBLIASIS]) |
| A07.2 | A07.2 (CRYPTOSPORIDIOSIS) |
| A07.3 | A07.3 (ISOSPORIASIS) |
| A07.4 | A07.4 (CYCLOSPORIASIS) |
| A07.8 | A07.8 (OTHER SPECIFIED PROTOZOAL INTESTINAL DISEASES) |
| A07.9 | A07.9 (PROTOZOAL INTESTINAL DISEASE, UNSPECIFIED) |
| A08.0 | A08.0 (ROTAVIRAL ENTERITIS) |
| A08.11 | A08.11 (ACUTE GASTROENTEROPATHY DUE TO NORWALK AGENT) |
| A08.19 | A08.19 (ACUTE GASTROENTEROPATHY DUE TO OTHER SMALL ROUND VIRUSES) |
| A08.2 | A08.2 (ADENOVIRAL ENTERITIS) |
| A08.31 | A08.31 (CALICIVIRUS ENTERITIS) |
| A08.32 | A08.32 (ASTROVIRUS ENTERITIS) |
| A08.39 | A08.39 (OTHER VIRAL ENTERITIS) |
| A08.4 | A08.4 (VIRAL INTESTINAL INFECTION, UNSPECIFIED) |
| A08.8 | A08.8 (OTHER SPECIFIED INTESTINAL INFECTIONS) |
| A09 | A09 (INFECTIOUS GASTROENTERITIS AND COLITIS, UNSPECIFIED) |
| R19.7 | R19.7 (DIARRHEA, UNSPECIFIED) |

## Table S2. Laboratory Testing related to Medically Attended Diarrhea (MAD) and *Clostridioides difficile*.

| **Test Name** | **Current Procedural Terminology (CPT) or Internal KP Code** | **Site** |
| --- | --- | --- |
| C DIFF PCR | . | KPNW |
| FINAL REPORT (VIBRIO) | 87046 | KPNW |
| CYCLOSPORA SMEAR, ACID FAST STAIN | 87206 | KPNW |
| MICROSPORIDIA SMEAR, STOOL | 87207 | KPNW |
| ISOSPORA SMEAR | 87207 | KPNW |
| CRYPTOSPORIDIUM, STOOL, DFA | 87272 | KPNW |
| CRYPTOSPORIDIUM ANTIGEN, EIA | 87328 | KPNW |
| GIARDIA LAMBLIA ANTIGEN, EIA | 87329 | KPNW |
| ENTAMOEBA HISTOLYTICA ANTIGEN | 87337 | KPNW |
| HELICOBACTER PYLORI ANTIGEN, STOOL | 87338 | KPNW |
| ROTAVIRUS AG STOOL | 87425 | KPNW |
| GI PARASITE PANEL (GIARDIA, CRYPTOSPORIDIUM, E. HISTOLYTICA), STOOL, MULTIPLEX PCR | 87505 | KPNW |
| BACTERIAL GI PANEL (SALMONELLA, SHIGELLA/EIEC, CAMPYLOBACTER, SHIGA TOXIN DNA), STOOL, MULTIPLEX PCR | 87505 | KPNW |
| GI VIRUS PANEL (ADENOVIRUS, ASTROVIRUS, NOROVIRUS, ROTAVIRUS, SAPOVIRUS), STOOL, MULTIPLEX PCR | 87505 | KPNW |
| GASTROINTESTINAL PATHOGEN PANEL (9 COMPONENTS), STOOL, MULTIPLEX PCR | 87506 | KPNW |
| EXTENDED GI PANEL (SALM, CAMPY, SHIG, STEC, PLESIO, VIB, ETEC, YERS), STOOL, MULTIPLEX PCR | 87506 | KPNW |
| OVA AND PARASITES EXAMINATION, CONCENTRATION, DIRECT SMEAR AND SPECIAL STAIN | 213091 | KPNW |
| PARASITE EXAM, STOOL, CONCENTRATION W DIRECT SMEAR AND STAIN | 218314 | KPNW |
| STOOL CULTURE (SALMONELLA, SHIGELLA, CAMPYLOBACTER, E. COLI 0157) | 221266 | KPNW |
| CLOSTRIDIUM DIFFICILE ANTIGEN AND TOXIN, A AND B, W REFLEX TO PCR | 231607 | KPNW |
| CYCLOSPORA AND CYSTOISOSPORA EXAMINATION, STOOL, ACID-FAST STAIN | 252329 | KPNW |
| O AND P IDENTIFICATION | 87177 | KPSC |
| CULTURE, ENTEROVIRUS RAPID SHELL VIAL | 87254 | KPSC |
| HELICOBACTER PYLORI ANTIGEN, STOOL | 87338 | KPSC |
| ROTAVIRUS | 87425 | KPSC |
| TOXINS, EIA WITH REFLEX TO E. COLI 157 CULTURE | 87427 | KPSC |
| BACTERIAL GI PANEL [SALMONELLA, SHIGELLA/EIEC, CAMPYLOBACTER, SHIGA TOXIN DNA], STOOL, MULTIPLEX PCR | 87505 | KPSC |
| CLOSTRIDIUM DIFFICILE, LIQUID STOOL, TOXIGENIC + NAP1 STRAIN, PCR | 247947 | KPSC |
| CLOSTRIDIUM DIFFICILE ANTIGEN AND TOXINS A AND B W REFLEX TO PCR | 87449, 87324 | KPSC |
| PARASITE STOOL SCREEN (GIARDIA SPP., CRYPTOSPORIDIUM SPP. AND/OR E. HISTOLYTICA ANTIGEN DETECTION WITH REFLEX TO PARASITE STOOL PCR CONFIRMATION AND DIFFERENTIATION) | 87449, 87505 | KPSC |

**Table S3. Charlson Comorbid Conditions and Associated ICD-10 Diagnosis Codes.**

| **Charlson Comorbid Condition** | **ICD codes from both inpatient and/or outpatient settings** |
| --- | --- |
| Myocardial infarction | **ICD-10**: I21.x, I22.x, I25.2 |
| Congestive heart failure | **ICD-10**: I09.9, I11.0, I13.0, I13.2, I25.5, I42.0, I42.5-I42.9, I43.x, I50.x, P29.0 |
| Peripheral vascular disease | **ICD-10**: I70.x, I71.x, I73.1, I73.8, I73.9, I77.1, I79.0, I79.2, K55.1, K55.8, K55.9, Z95.8, Z95.9 |
| Cerebrovascular disease | **ICD-10**: G45.x, G46.x, H34.0, I60.x-I69.x |
| Dementia | **ICD-10**: F00.x-F03.x, F05.1, G30.x, G31.1 |
| Chronic pulmonary disease | **ICD-10**: I27.8, I27.9, J40.x-J47.x, J60.x-J67.x, J68.4, J70.1, J70.3 |
| Peptic ulcer disease | **ICD-10**: K25.x-K28.x |
| Mild liver disease | **ICD-10**: B18.x, K70.0-K70.3, K70.9, K71.3-K71.5, K71.7, K73.x, K74.x, K76.0, K76.2-K76.4, K76.8, K76.9, Z94.4 |
| Diabetes (with and without chronic complication) | **ICD-10**: E10.0-E10.9, E11.0-E11.9, E12.0-E12.9, E13.0-E13.9, E14.0- E14.9 |
| Hemiplegia or paraplegia | **ICD-10**: G04.1, G11.4, G80.1, G80.2, G81.x, G82.x, G83.0-G83.4, G83.9 |
| Renal Disease | **ICD-10**: I12.0, I13.1, N03.2-N03.7, N05.2-N05.7, N18.x, N19.x, N25.0, Z49.0-Z49.2, Z94.0, Z99.2 |
| Malignancy, including leukemia and lymphoma (≠ malignant neoplasm of skin) | **ICD-10**: C00.x-C26.x, C30.x-C34.x, C37.x-C41.x, C43.x, C45.x-C58.x, C60.x-C76.x, C81.x-C85.x, C88.x, C90.x-C97.x |
| Moderate or severe liver disease | **ICD-10**: I85.0, I85.9, I86.4, I98.2, K70.4, K71.1, K72.1, K72.9, K76.5, K76.6, K76.7 |
| Metastatic solid tumor | **ICD-10**: C77.x-C80.x |
| Organ Transplant | Identified by National Transplant Services Directory |
| AIDS/HIV | Identified by HIV Registry |

**Table S4. Testing for *Clostridioides difficile* infection (CDI) among medically-attended diarrhea (MAD) episodes, by age and race/ethnicity, 2016-2021**

|  | **Non-Hispanic Asian** | **Non-Hispanic Black** | **Hispanic** | **Pacific Islander/Other** | **Unknown** | **Non-Hispanic White** | **Total** |
| --- | --- | --- | --- | --- | --- | --- | --- |
|  | N (%) | | | | | | |
| **All Ages** |  |  |  |  |  |  |  |
| Total MAD Episodes | 49,379 | 64,474 | 314,499 | 19,005 | 16,482 | 313,694 | 777,533 |
| CDI laboratory test ordered | 5,211 (10.6) | 7,653 (11.9) | 30,654 (9.8) | 1,951 (10.3) | 1,015 (6.2) | 47,480 (15.1) | 93,964 (12.1) |
| **18-49 years** |  |  |  |  |  |  |  |
| Total MAD Episodes | 25,438 | 33,029 | 198,556 | 11,873 | 12,532 | 126,468 | 407,896 |
| CDI laboratory test ordered | 2,004 (7.9) | 2,641 (8.0) | 15,090 (7.6) | 955 (8.0) | 638 (5.1) | 12,706 (10.1) | 34,034 (8.3) |
| **50-69 years** |  |  |  |  |  |  |  |
| Total MAD Episodes | 15,652 | 20,995 | 83,748 | 5,056 | 3,332 | 103,128 | 231,911 |
| CDI laboratory test ordered | 1,721 (11.0) | 2,824 (13.5) | 9,341 (11.2) | 635 (12.6) | 298 (8.9) | 16,631 (16.1) | 31,450 (13.6) |
| **70+ years** |  |  |  |  |  |  |  |
| Total MAD Episodes | 8,289 | 10,450 | 32,195 | 2,076 | 618 | 84,098 | 137,726 |
| CDI laboratory test ordered | 1,486 (17.9) | 2,188 (20.9) | 6,223 (19.3) | 361 (17.4) | 79 (12.8) | 18,143 (21.6) | 28,480 (20.7) |

**Table S5. Rate of testing for *Clostridioides difficile* infection (CDI) among medically-attended diarrhea (MAD) episodes, 2016-2021**

|  | **Overall** | | | |
| --- | --- | --- | --- | --- |
|  | **Count of CDI Tests** | **Person-years** | **Testing Rate (per 100,000 PY)** | **95% CI** |
| **Year** |  |  |  |  |
| 2016 | 23,728 | 3,605,900 | 658.0 | 649.7, 666.5 |
| 2017 | 25,131 | 3,768,964 | 666.8 | 658.6, 675.1 |
| 2018 | 26,504 | 3,915,582 | 676.9 | 668.8, 685.1 |
| 2019 | 27,086 | 3,992,901 | 678.4 | 670.3, 686.5 |
| 2020 | 21,721 | 4,085,657 | 531.6 | 524.6, 538.8 |
| 2021 | 25,354 | 4,121,364 | 615.2 | 607.6, 622.8 |
